# Non-invasive prenatal testing reveals copy number variations related to pregnancy complications

**DOI:** 10.1186/s13039-019-0451-3

**Published:** 2019-08-30

**Authors:** Guangping Wu, Rong Li, Chao Tong, Miaonan He, Zhiwei Qi, Huijuan Chen, Tao Deng, Hailiang Liu, Hongbo Qi

**Affiliations:** 1 Department of Obstetrics, The First Affiliated Hospital of Chongqing Medical University, Chongqing, 400016 People’s Republic of China; 2 State Key Laboratory of Maternal and Fetal Medicine of Chongqing Municipality, Chongqing Medical University, Chongqing, 400016 People’s Republic of China; 3 International Collaborative Laboratory of Reproduction and Development of Chinese Ministry of Education, Chongqing Medical University, No.1 Youyi Road, Yuzhong District, Chongqing, 400016 People’s Republic of China; 4 Beijing CapitalBio Medical Laboratory, Beijing, 101111 China; 5 CapitalBio Technology Inc., Beijing, 101111 China; 6 Department of Medical Genetics, School of Basic Medical Sciences, Southern Medical University, Guangzhou, 510515 Guangdong China

**Keywords:** Noninvasive prenatal testing (NIPT), Copy number variation (CNV), Gestational diabetes mellitus (GDM), Pregnancy-induced hypertension (PIH), Preterm prelabor rupture of membranes (PPROM), Placenta implantation abnormalities (PIA)

## Abstract

**Background:**

Pregnancy complications could lead to maternal and fetal morbidity and mortality. Early diagnosing and managing complications have been associated with good outcomes. The placenta was an important organ for development of pregnancy complications. Thus, non-invasive prenatal testing technologies could detect genetic variations, such as aneuploidies and sub-chromosomal copy number variations, reflecting defective placenta by maternal plasma cffDNAs. Maternal cffDNAs had been proved to derive from trophoblast cells of placenta.

**Results:**

In order to find out the relationship between genetic variations and pregnancy complications, we reviewed NIPT results for subchromosomal copy number variations in a cohort of 3890 pregnancies without complications and 441 pregnancies with pregnancy complications including gestational diabetes mellitus (GDM), pregnancy-induced hypertension (PIH), preterm prelabor rupture of membranes (PPROM) and placenta implantation abnormalities (PIA). For GDMs, we identified three CNV regions containing some members of alpha- and beta-defensins, such as DEFA1, DEFA3, DEFB1. For PIHs, we found three duplication and one deletion region including Pcdhα, Pcdhβ, and Pcdhγ, known as protocadherins, which were complicated by hypertensive disorders. For PPROMs and PIAs, we identified one and two CNV regions, respectively. SFTPA2, SFTPD and SFTPA1, belonging to surfactant protein, was considered to moderated the inflammatory activation within the fetal extra-embryonic compartment, associated to duration of preterm prelabor rupture of fetal membranes, while MEF2C and TM6SF1 could be involved in trophoblast invasion and differentiation.

**Conclusions:**

Our findings gave a clue to correlation between genetic variations of maternal cell-free DNAs and pregnancy complications.

**Electronic supplementary material:**

The online version of this article (10.1186/s13039-019-0451-3) contains supplementary material, which is available to authorized users.

## Background

Pregnancy complications affects maternal and fetal health. Medical diseases such as hypertension and diabetes during pregnancy, as well as the poor delivery conditions (placenta accrete and prelabor rupture of membranes) were common complications in pregnancy [1]. The major challenges lies in optimizing earlier predictors and identifiers of pregnancy complications. Delays in diagnosing and managing complications have been associated with poor outcomes [2]. Shortening the time between the onset of a complication and the initiation of appropriate management enables prevention and reduction of maternal and fetal morbidity and mortality [3]. However, the value of screening those with pregnancy complications is uncertain because of limitations in knowledge on their contribution to such pregnancy complications and lack of evidence for effective intervention.

The placenta is an important organ for human reproduction. It is shared by both mother and fetus and is responsible for oxygen and nutrient supply and waste elimination from the growing fetus [4]. Multi-omic studies are required to more fully understand the molecular changes that affect the placenta and associated pregnancy. Biofluids have also been analyzed to characterize differences in the fetoplacental and maternal metabolites in pregnancies with poor outcomes compared to normal pregnancies [5]. Defective placentation, particularly following poor trophoblast invasion, predisposes to a continuum of pregnancy complications in which genetic and environmental factors may interact to determine the timing and severity of disease [6].

A rapidly evolving field in prenatal diagnosis is non-invasive prenatal testing (NIPT), also referred to as non-invasive prenatal screening (NIPS). NIPT is based on the detection of cell-free fetal DNA in maternal plasma using next-generation sequencing or other methods for fetal DNA assessment, mainly used for detection of three common aneuploidies (13, 18, 21) [7]. However, the technology is already being refined to detect genome-wide microdeletion/duplication [8], which have been identified as a common cause of a number of human diseases.

Here, we reviewed NIPT results of screening for subchromosomal microdeletions and microduplications within a cohort of 3890 pregnancies without complications and 441 pregnancies with pregnancy complications, including Gestational diabetes mellitus (GDM), pregnancy-induced hypertension (PIH), preterm prelabor rupture of membranes (PPROM) and placenta implantation abnormalities (PIA). Based on NIPT, CNVs were detected by cff-DNA in maternal plasma, derived from trophoblast cell of placenta, thus, we could give some evidences for acossiation existed between CNVs and risks to develop pregnancy complications.

## Results

### Subject

From October 2017 to July 2018, there were 3890 pregnancies without complications and 441 pregnancies with one of the four types of pregnancy complications and were undergoing NIPT. Pregnant women with the four types of pregnancy complications, including 177 cases with gestational diabetes mellitus, 28 cases with pregnancy-induced hypertension, 173 cases with preterm prelabor rupture of membranes and 63 cases with placenta implantation (Table 1). All of subjects were undergoing NIPT on gestational age from 12 to 24 weeks, prenatal diagnosis and delivering in the hospital. The maternal age for the 441 pregnancies with pregnancy complications was 29.36 ± 4.15 years old and for the 3890 pregnancies without complications was 29.62 ± 4.28 years old in average. In addition, we identified cases with chromosomal microdeletions or microduplications. Overall, we found 1082 (27.81%) cases with chromosomal microdeletions or microduplications in pregnancies without pregnancy complications group, while we found 121 (27.81%) cases with chromosomal microdeletions or microduplications in pregnancies with pregnancy complications group, including 51 (28.81%) cases with gestational diabetes mellitus, 5 (17.86%) cases with pregnancy-induced hypertension, 44 (25.43%) cases with preterm prelabor rupture of membranes and 11 (17.46%) cases with placenta implantation.

**Table 1:** The type of pregnant women with pregnancy complications

The type of pregnancy complications	The number of cases
Preterm Prelabor rupture of membranes	173
Placenta implantation abnormalities	63
Pregnancy-induced hypertension	28
Gestational diabetes mellitus	177
Normal	3890

### Gestational diabetes mellitus

**Fig. 1:**
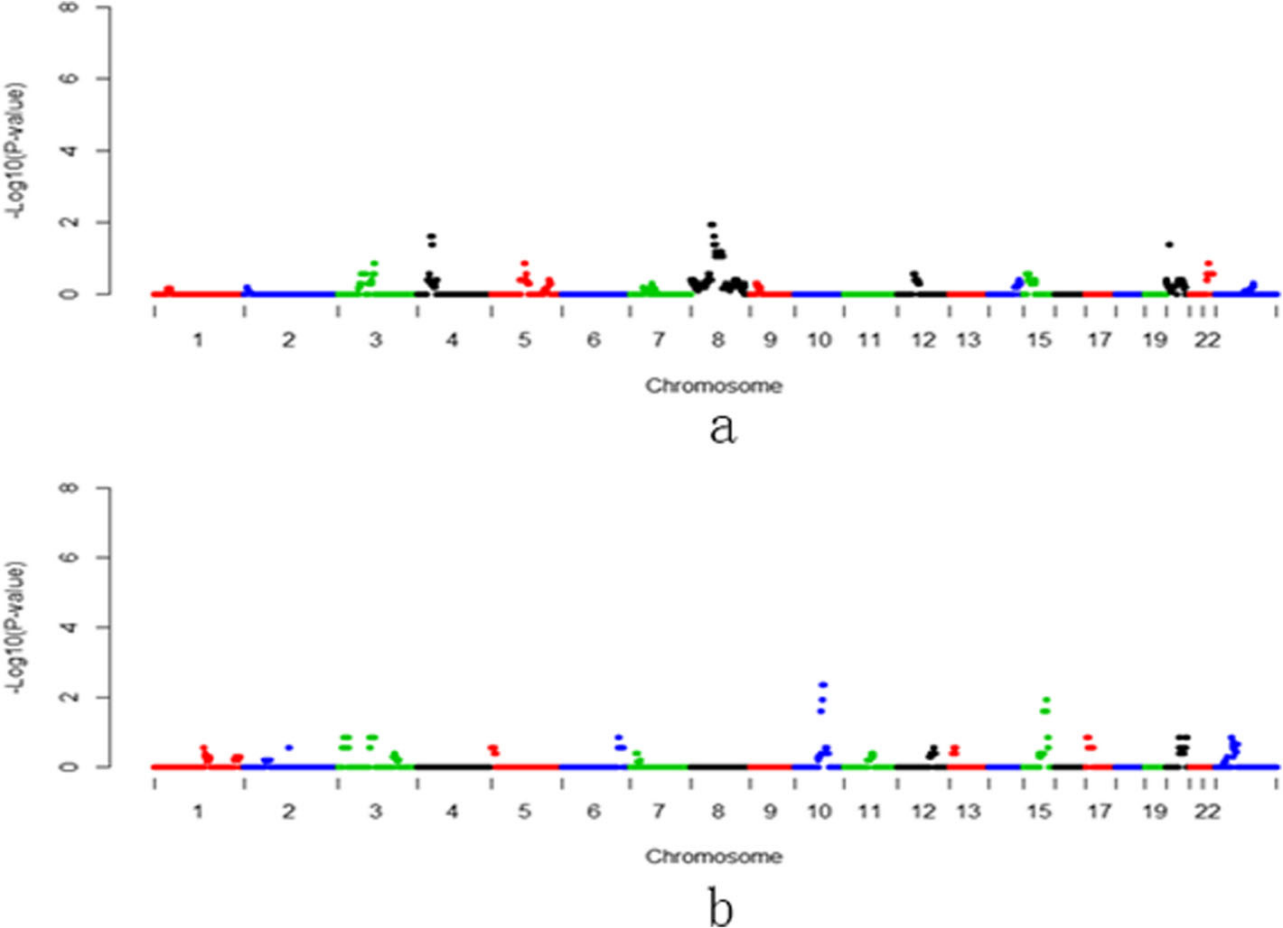
Manhattan plots of 1 Mb-bin on NIPT-CNVs for Gestational diabetes mellitus (GDM). **a**: duplication region. **b**: deletion region

Gestational diabetes mellitus (GDM) is defined as glucose intolerance diagnosed during pregnancy [9]. GDM increases risk of adverse pregnancy outcomes and has substantial long-term adverse health impacts on both mothers and their offspring, including a predisposition to ischemic heart disease, hypertension, obesity, metabolic syndrome and type 2 diabetes mellitus (T2DM) in later life [10, 11]. Despite years of investigation, very little is known about the genetic predisposition for gestational diabetes mellitus (GDM). Increasing number of studies have reported pathological changes of placenta tissues in gestational diabetes mellitus (GDM), while the underlying mechanisms involved in this process are still largely uncertain [12, 13]. We herein tried to identify the genetic variation of placenta involving GDM currencies by the cell-free DNA in the maternal plasma, which was proved to be derived from placental trophoblast cells [14, 15]. As a result, we found three chromosomal regions of *p*-value < 0.01 were considered as high risks in currencies of GDM (Fig. 1, Table 2 and Additional file 1: Table S1). Then, enrichment analysis for 151 genes on the three regions was performed using DAVID website. Gene Ontology enrichment showed that defense response to bacterium (GO:0042742), innate immune response (GO:0045087), killing of cells of other organism (GO:0031640), antibacterial humoral response (GO:0019731) and defense response to fungus (GO:0050832) were the top five significant GO terms in Biological Process catalog (Additional file 1: Table S2). However, most of enriched genes were from alpha-defensins and beta-defensins families (also see Additional file 1: Table S2). Alpha-defensins display chemotactic activity and induce proinflammatory cytokines [16]. It can be released into the extracellular milieu following granulocyte activation as a consequence of degranulation, leakage, cell death, and lysis during inflammation [17]. Meanwhile, in addition to their antibacterial and antiviral effects, beta-defensins may play roles in the range of protective, adhesive and regulatory functions [18]. Moreover, several studies [19–23] reported the associations between the type 1 and 2 diabetes and gene copy number as well as polymorphism and mRNA expression on some members of alpha- and beta-defensins, such as DEFA1, DEFA3, DEFB1, which occurred in our gene list.

**Table 2:** CNVs and genes associated wiith Gestational diabetes mellitus (GDM), pregnancy-induced hypertension (PIH), preterm prelabor rupture of membranes (PPROM) and placenta implantation abnormalities (PIA)

Pregnant complicatons	dup/del	Genome Region	Gene Numer
Gestational diabetes	dup	Chr1:26-29 Mb	67
	dup	Chr8:1-16 Mb	71
	dup	ChrX:75-78 Mb	13
Pregnancy-induced hypertension	dup	Chr3:1-2 Mb	1
	dup	Chr3:149-151 Mb	18
	dup	Chr11:82-91 Mb	34
	dup	Chr12:50-73 Mb	284
	del	Chr5:138-150 Mb	157
Premature rupture of membranes	del	Chr10:80-86 Mb	24
Placenta implantation	dup	cht5:88-90 Mb	6
	del	chr15:83-84 Mb	13

### Pregnancy-induced hypertension

**Fig. 2:**
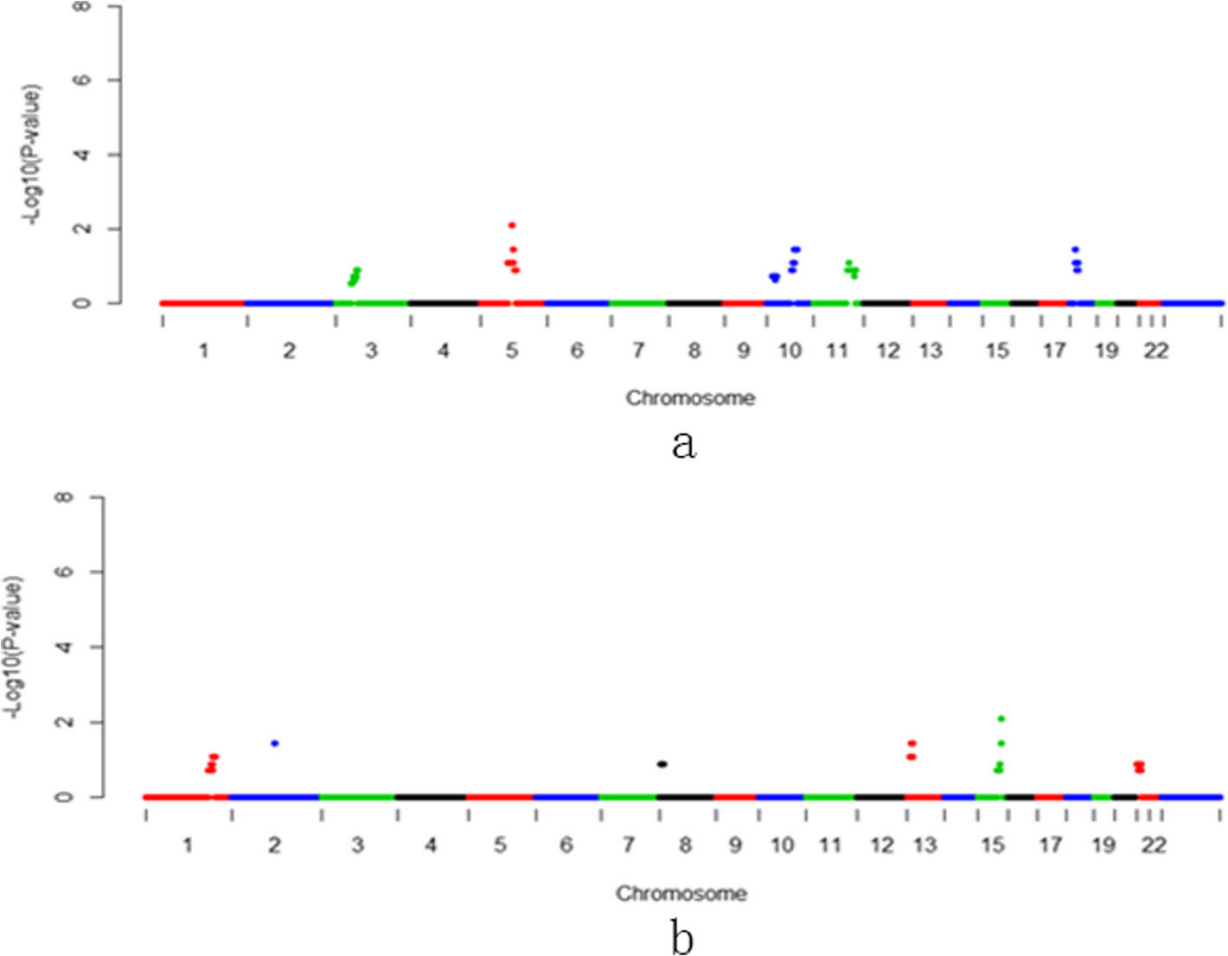
Manhattan plots of 1 Mb-bin on NIPT-CNVs for pregnancy-induced hypertension (PIH). **a**: duplication region. **b**: deletion region

Hypertension is one of the most common complication during pregnancy. It contributes significantly to maternal and perinatal morbidity and mortality. Placental insufficiency is believed to be a mechanism of pregnancy-induced hypertension (PIH) [24]. Placental hypoxia is believed to result in the release of a variety of placental factors that have profound effects on blood flow and arterial pressure regulation [25]. Several studies have been performed to assess the validity of uterine Doppler examinations as a screening tool for PIH [26]. We herein identified three duplication and one deletion of *p*-value < 0.01, considered as high risks in currencies of PIH (Fig. 2, Table 2 and Additional file 1: Table S1). Then, Gene Ontology enrichment for 494 genes on the four regions showed the top five significant GO terms in Biological Process catalog were homophilic cell adhesion via plasma membrane adhesion molecules (GO:0007156), calcium-dependent cell-cell adhesion via plasma membrane cell adhesion molecules (GO:0016339), nervous system development (GO:0007399), negative regulation of serine-type endopeptidase activity (GO:1900004), and synapse assembly (GO:0007416). (Additional file 1: Table S3). However, most of enriched genes were from protocadherins (also see Additional file 1: Table S3). Protocadherin have three gene clusters in human, including Pcdhα, Pcdhβ, and Pcdhγ, located on the 5q31 region of Chromosome 5 [27]. Some studies were proved that some members of protocadherins orthologs deficiency could alters development, morphogenesis and transcriptional profile of the placenta in mouse [28, 29]. Protocadherins were also expressed prominently by developing blood vessels during angiogenesis [30]. Thus, deficiency protocadherins could lead placental lesions, which were defined as maternal vascular lesions, were more common in pregnancies that were complicated by hypertensive disorders [31], although PIH is primarily an impairment of the maternal circulatory system that is believed to be the result of early developmental events that lead to inadequate vascular remodeling and/or structural abnormalities of maternal arteries [32].

### Preterm prelabor rupture of membranes

**Fig. 3:**
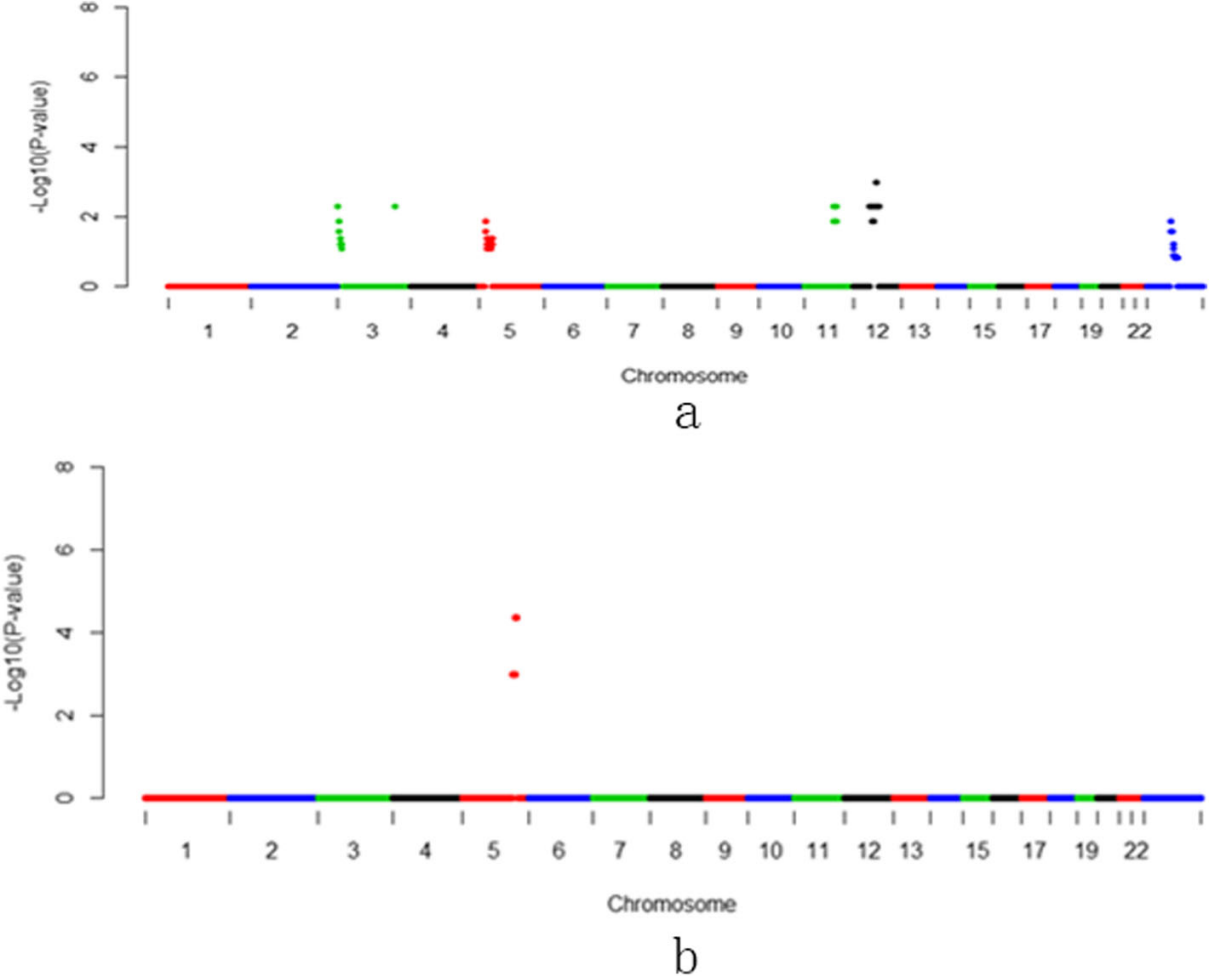
Manhattan plots of 1 Mb-bin on NIPT-CNVs for preterm premature rupture of membranes (PPRM). **a**: duplication region. **b**: deletion region

Preterm prelabor rupture of membranes (PPROM), defined as rupture of the amnion and corium before the onset of labor, responsible for approximately one-third of Prelabor births and 3% of all pregnancies [33]. The most prevalent site of rupture of amniotic membranes in PPROM is the supra-cervical area (membrane overlying the ostium of cervical area). The amniotic membrane at this site is structurally altered and easily disrupted, associated with marked swelling and disruption of the collagen network within the compact, fibroblast and spongy layers [34]. Approximately of PPROM was involved by microbial invasion of the amniotic cavity [35]. We herein identified one deletion region of *p*-value < 0.01, considered as high risks in currencies of PPROM (Fig. 3, Table 2 and Additional file 1: Table S1). Then, Gene Ontology enrichment for 24 genes on the region showed the significant GO terms in Biological Process catalog were respiratory gaseous exchange (GO:0007585). (Additional file 1: Table S4) There were three genes locating on this region, including SFTPA2, SFTPD and SFTPA1. The proteins encoded by this gene belonging to surfactant protein, is part of the innate immune response, against inhaled microorganisms and chemicals, involved in surfactant metabolism [36]. Several studies revealed fetal surfactant proteins (Surfactant protein-A, Surfactant protein-C and Surfactant protein-D) moderate the inflammatory activation within the fetal extra-embryonic compartment, associated to duration of preterm Prelabor rupture of fetal membranes [37–39].

### Placenta implantation abnormalities

**Fig. 4:**
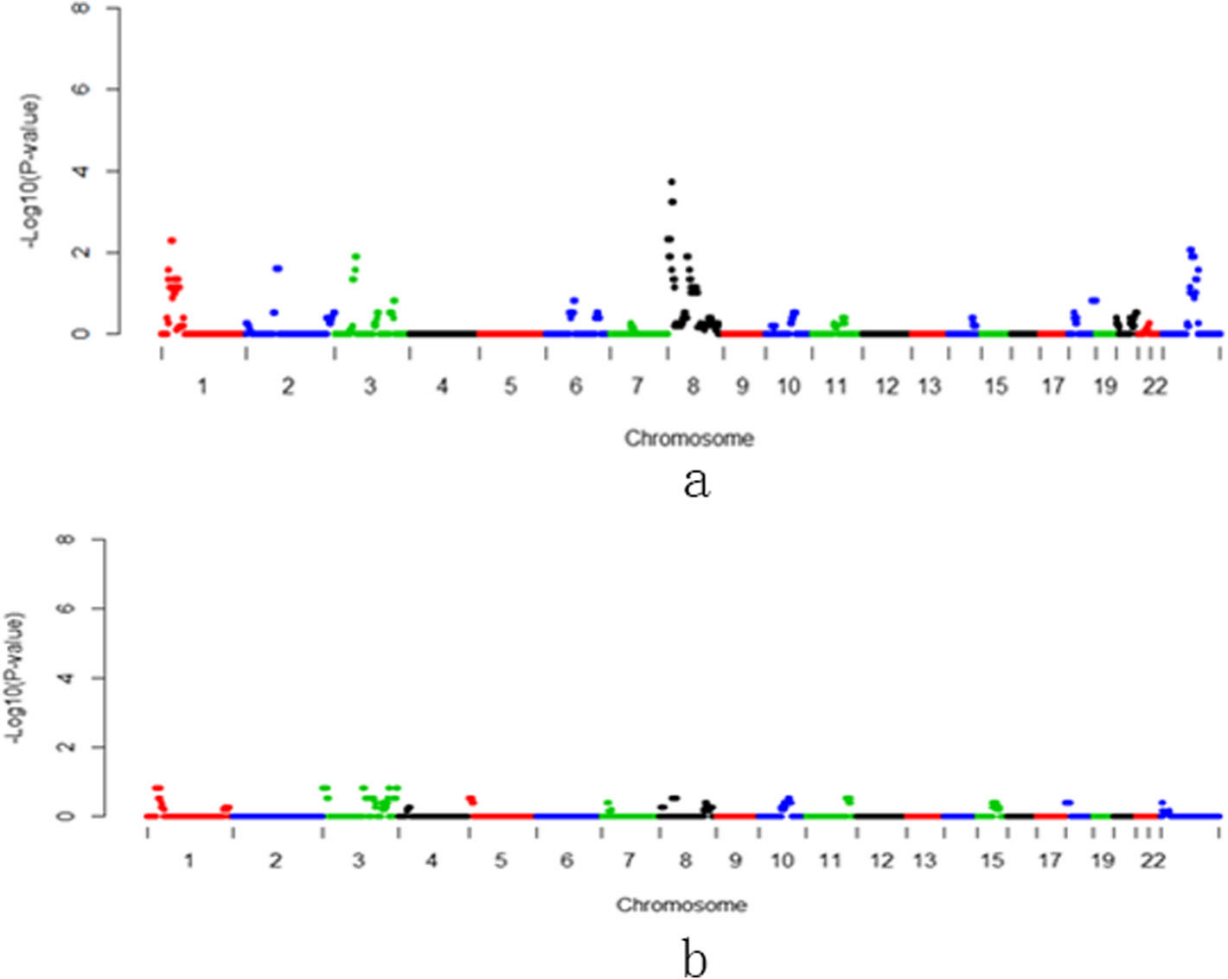
Manhattan plots of 1 Mb-bin on NIPT-CNVs for placenta implantation abnormalities (PIA). **a**: duplication region. **b**: deletion region

Placental implantation abnormalities (PIAs), including placenta previa, placenta accreta, vasa previa, and velamentous cord insertion, can have catastrophic complications for both the mother and fetus, strongly associated to preterm delivery resulting in significant perinatal morbidity and mortality [40]. During the process of implantation, fetal trophoblast cells invade and migrate into the maternal decidua and destroy the wall of the maternal spiral arteries, converting them from muscular vessels into flaccid sinusoidal sacs [41]. Trophoblast differentiation along the invasion is fundamental to early implantation, placental development and establishment of the fetal-maternal interface. Multiple gene pathways and heterogeneous cellular interactions have been described in directing and controlling trophoblast invasion [42–44]. We herein identified one deletion region and one duplication region of *p*-value < 0.01, considered as high risks in currencies of PIAs (Fig.4, Table 2, and Additional file 1: Table S1). Then, 19 genes on the regions were annotated. Although there was not any enrichment for pathways and gene ontologies, some genes were reported to be involved in trophoblast invasion and differentiation, such as MEF2C [45] and TM6SF1 [46], both of which were supposed to be the key genes on the duplication/deletion region, respectively. MEF2C (myocyte-specific enhancer factor 2C), activated by Mekk3, was a transcription factor crucial for early embryonic cardiovascular development through the p38 mitogen-activated protein kinase (Mapk) cascade [47]. Placental expression of TM6SF1 appears to be upregulated by environmental exposures such as prenatal alcohol consumption [48]. TM6SF1 (transmembrane 6 superfamily member 1) could be regulated by DNA methylation, potentially facilitating protein trafficking via organelle fusion.

## Discussion

There are various theories to provide a better understanding of the potential mechanisms responsible for the pathogenesis of pregnancy complications, which include genetic predisposition, imbalance of immune system, placenta shallow implantation, vascular endothelium damage, ischemia and hypoxia of placenta. The outcomes of most pregnancy complications are unpredictable unless they are managed by appropriate health care providers. Previous studies have identified factors predicting the care seeking behaviors for pregnancy complications include higher educational status, near distance to health facility, availability of transport system, small family size, previous pregnancy experience, and good economic status, that have been observed with high probability of skilled assistance seeking for pregnancy complications [49].

In order to find out the relationship between genetic variations and pregnancy complications, we used NIPT to screen a large population in the Chongqing area. This NIPT technology uses a semiconductor sequencing platform (SSP) to reliably detect CNV in pregnancies carrying high-risk fetuses. Here, we reviewed NIPT results in the context of screening for subchromosomal microdeletions and microduplications within a cohort of 441 pregnancies with pregnancy complications and the 3890 pregnancies without complications. To study whether maternal serum markers tested as a part of screening additionally help in predicting other pregnancy outcomes including gestational diabetes mellitus (GDM), pregnancy-induced hypertension (PIH), preterm Prelabor rupture of membranes (PPRM) and placenta implantation abnormalities (PIA).

This study was designed to test the hypothesis whether a direct correlation exists between sub-chromosomal microdeletions and microduplications on placenta and pregnancy complications. We had demonstrated the feasibility of performing noninvasive prenatal detection of fetal chromosomal microdeletions and microduplications on a genome-wide level and at 3 Mb resolution [8]. Not all CNVs, however, are disease-causing, while some CNVs have been identified in apparently normal individuals. Whether a CNV is disease-causing or not depends on many factors, such as gene content (e.g., a CNV that is gene-rich is more likely to cause a phenotype than one containing few or no genes). Therefore, understanding the effects of CNVs on pregnancy complications is important in clinical medicine. Herein, we identified which CNVs cause a clinical phenotype versus those that are part of normal variation. Further, the mechanism was not well-understood for potential disease-associated CNVs loci in our study, possibly due to epigenetic modifications, other genetic variants in the vicinity region, modifier genes and regulatory elements, [50] and potentially also due to allele dosage effects in combination with alternative allelic copies present within the CNV regions [51].

Placenta is essential for maintenance of pregnancy and for promoting normal growth and development of fetus. Placenta formation and function can affect fetal survival, growth, and development and can modulate maternal immune responses [52]. The inner cell mass of the blastocyst gives rise to the embryo, while the outer cell layer, the trophectoderm, gives rise to the placenta. Maternal-fetal cellular trafficking (MFCT) is the bidirectional passage of cells between mother and fetus during pregnancy. This results in the presence of fetal cells in the maternal circulation, known as fetal microchimerism, and maternal cells in the fetal circulation, known as maternal microchimerism [15]. The biologic role of this bidirectional passage of cells during pregnancy has been implicated in development of the fetal immune system, tolerance mechanisms during pregnancy, tissue repair in autoimmune disease and cancer, and immune surveillance [53]. Clinical utility of MFCT could be used for prediction of pregnancy complications. Additionally, this transplacental passage of cells has been implicated in the delicate balance between immunologic priming and tolerance which can influence the occurrence of autoimmune disease and transplantation outcomes [54]. The DNAs derived from placenta could be detected by NIPT technologies. Both of aneuplodies and CNVs have been identified. In this study, we reanalyzed the assocications between NIPT data and clinical complications of pregnancy and fetus. The results indicated that some CNVs may be corelated to complications, such as gestational diabetes mellitus (GDM), pregnancy-induced hypertension (PIH), preterm prelabor rupture of membranes (PPROM) and placenta implantation abnormalities (PIA). It suggested that NIPT method would give a clue to investigate the correlation between CNVs and complications.

However, some limitations occurred in this study. Larger-scale analytical and functional investigations of CNVs contributing to pregnancy-related complications are still need to be performed to understand the molecular and cellular mechanisms underpinning. Moreover, low read-depth of NIPT and small samples of positive finding associated with maternal complications were also weak points in this study. Increasing read-depth of NIPT could give higher resolutions for CNVs detection [8]. Meanwhile, more positive samples were needed to be collected for proving the associations and investigating new findings for maternal complications in future.

## Conclusion

It is well established in the literature that abnormal combined and quadruple screen analytes, not explained by either maternal or fetal factors, are associated with a multitude of adverse pregnancy outcomes. This would allow closer monitoring of women identified to be at increased risk and identify candidates for participation in early intervention trials.

## Materials and methods

### Patients

From October 2017 to July 2018, 441 pregnant women with pregnancy complications and 3890 pregnant women without complications (The First Affiliated Hospital of Chongqing Medical University) opted for NIPT to avoid fetal T13, T18 and T21 aneuploidies. Complications inclusion criteria: ① Diagnostic criteria for gestational hypertension syndrome: systolic pressure 140 mmHg, or diastolic pressure 90 mmHg; ②Gestational diabetes mellitus (GDM) diagnosis: According to the WHO recommended standard, 75 g glucose tolerance test (OGTT) results confirmed (fasting glucose≥5.1 mmol/L), 1 h postprandial blood glucose ≥10.0 mmol/L, 2 h postprandial blood glucose ≥8. 5 L); or random detection of blood glucose ≥11.1 mmol/L, combined with three more and one less (eat more, drink more water, urine more, weight loss), was diagnosed as typical diabetes clinical manifestations.

### NIPT processing

Whole blood samples of 5 to 10 mL from pregnant women were collected in EDTA within 8 h or cell-free DNA was collected in BCT tubes (Streck Inc.; Omaha, NE) within 72 h at 4 °C. Afterwards, cfDNA extraction, library construction, quality control and pooling were performed according to the JingXin Fetal Chromosome Aneuploidy (T21, T18, T13) Testing Kits (CFDA registration permit No. 0153400300). Following the DNA sequencing, 15~20 libraries were pooled and sequenced within ~ 200 bp reads using the JingXin BioelectronSeq 4000 System (CFDA registration permit NO. 20153400309), which is a type of semiconductor sequencer. Sequencing reads were filtered and aligned to the human reference genome (hg19). Fetal DNA concentration was calculated as a quality control using our previously described method [8]. Combined GC-correction and Z-score testing methods were used to identify fetal autosomal aneuploidies, as described previously [55]. Meanwhile, fetal and maternal chromosome copy number variations (CNVs) were classified with our modified Stouffer’s Z-score method as described previously [8]. In a previous study, a cutoff value of Z-score > 3 was used to determine whether the ratio of the chromosomes was increased, and if fetal trisomies 21, 18, and 13 were also present. Here, each chromosome with an absolute value of the Z- score greater than 3 was marked as microduplications, while less than − 3 marked as microdeletion.

## Additional file

Additional file 1: **Table S2.** Enrichment analysis of gene ontology for CNVs in gestational diabetes mellitus. **Table S3.** Enrichment analysis of gene ontology for CNVs in pregnancy-induced hypertension. **Table S4.** Enrichment analysis of gene ontology for CNVs in preterm prelabor rupture of membranes. (XLSX 66 kb)

## Data Availability

The datasets used and/or analyzed during the current study are available from the corresponding author on reasonable request.

## Abbreviations

cfDNA: cell-free DNA
NGS: Next generation sequencing
NIPT: Non-invasive prenatal testing
PD: Prenatal diagnosis
